# Chemokines as a Conductor of Bone Marrow Microenvironment in Chronic Myeloid Leukemia

**DOI:** 10.3390/ijms18081824

**Published:** 2017-08-22

**Authors:** Naofumi Mukaida, Yamato Tanabe, Tomohisa Baba

**Affiliations:** Division of Molecular Bioregulation, Cancer Research Institute, Kanazawa University, Kakuma-machi, Ishikawa, Kanazawa 920-1192, Japan; t.yamato@stu.kanazawa-u.ac.jp (Y.T.); sergenti@staff.kanazawa-u.ac.jp (T.B.)

**Keywords:** CCL3, CXCL12, CXCR4, hematopoietic stem cell, hematopoietic progenitor cell, leukemia stem cell, niche

## Abstract

All blood lineage cells are generated from hematopoietic stem cells (HSCs), which reside in bone marrow after birth. HSCs self-renew, proliferate, and differentiate into mature progeny under the control of local microenvironments including hematopoietic niche, which can deliver regulatory signals in the form of bound or secreted molecules and from physical cues such as oxygen tension and shear stress. Among these mediators, accumulating evidence indicates the potential involvement of several chemokines, particularly CXCL12, in the interaction between HSCs and bone marrow microenvironments. Fusion between *breakpoint cluster region* (*BCR*) and *Abelson murine leukemia viral oncogene homolog* (*ABL*)*-1* gene gives rise to BCR-ABL protein with a constitutive tyrosine kinase activity and transforms HSCs and/or hematopoietic progenitor cells (HPCs) into disease-propagating leukemia stem cells (LSCs) in chronic myeloid leukemia (CML). LSCs can self-renew, proliferate, and differentiate under the influence of the signals delivered by bone marrow microenvironments including niche, as HSCs can. Thus, the interaction with bone marrow microenvironments is indispensable for the initiation, maintenance, and progression of CML. Moreover, the crosstalk between LSCs and bone marrow microenvironments can contribute to some instances of therapeutic resistance. Furthermore, evidence is accumulating to indicate the important roles of bone marrow microenvironment-derived chemokines. Hence, we will herein discuss the roles of chemokines in CML with a focus on bone marrow microenvironments.

## 1. Introduction

Hematopoietic stem cells (HSCs), the only cells capable of producing all blood cell lineages, are present in diverse tissues throughout development, beginning in the aorta-gonad-mesonephron region and the yolk sac followed by the placenta, fetal liver, spleen, and bone marrow [1]. After birth, the bone marrow is the primary site of hematopoiesis, which occurs through the orchestrated proliferation, self-renewal, and differentiation of HSCs, followed by egress of mature progeny into the circulating blood. HSCs are often vulnerable to mutagenesis arising from attenuated DNA repair and DNA damage responses, but DNA damage can activate p53, thereby inhibiting cell cycle to promote quiescence in damaged HSCs and to induce apoptosis of genetically injured HSCs [2]. Consequently, p53 can maintain the integrity of HSC pool in a cell-autonomous manner. In addition to p53, HSC fate is also controlled by local microenvironments including niche, which can deliver regulatory signals in the form of bound or secreted molecules and from physical cues such as oxygen tension and shear stress [1]. Among the regulatory signals, the interaction between a chemokine, CXCL12, and its receptor, CXCR4, has emerged as an important regulator of normal hematopoiesis [1,3].

Genetic events occur in self-renewing HSCs in the case of chronic phase chronic myeloid leukemia (CML) and most cases of acute myeloid leukemia (AML), or in early hematopoietic progenitor cells (HPCs) in a proportion of AML, such as granulocyte-macrophage progenitors in and a granulocyte-macrophage progenitor-like subset in blast crisis CML [4]. Nevertheless, such genetic events result in the generation of the disease-propagating leukemia cells, referred to as leukemic stem cells (LSCs), which can self-renew, proliferate and differentiate, as can normal HSCs. Evidence is accumulating to indicate that LSCs needs the signals delivered by bone marrow microenvironments including niche cells for their initiation, maintenance, and proliferation [4]. Among the signals, the CXCL12–CXCR4 axis can be an important signal pathway in local environment-mediated leukemogenesis [4]. Moreover, the crosstalk between LSCs and bone marrow microenvironments is presumed to be involved in some instances of therapeutic resistance [5]. Thus, the interaction between LSCs and bone marrow microenvironments can be a novel target to treat CML.

Here, after briefly reviewing normal hematopoiesis, CML, and chemokines in normal hematopoiesis, we will discuss the roles of chemokines in CML.

## 2. Results

### 2.1. Normal Hematopoiesis and Niche

Like the stem cells in other organs, HSCs under resting conditions divide in an asynchronous manner to keep the total pool of HSCs approximately at the same levels [6]. Accordingly, an HSC undergoes simultaneously a process of self-renewal and one of differentiation down the committed progenitor pathway. Until now, important insights have been made to identify the molecules involved in this stem cell fate, including CXCL12 [7], stem cell factor (SCF) [8], Notch [9], Sonic hedgehog [10], Wnt [11], and transforming growth factor (TGF)-β [12]. These molecules are produced mostly by non-hematopoietic cells present in bone marrow microenvironment, together with additional secreted molecules and those expressed on cell surface. Thus, synthetic culture environment cannot completely recapitulate bone marrow microenvironment; consequently, a large-scale ex vivo expansion of HSCs has been very difficult to succeed by present.

**Figure 1:**
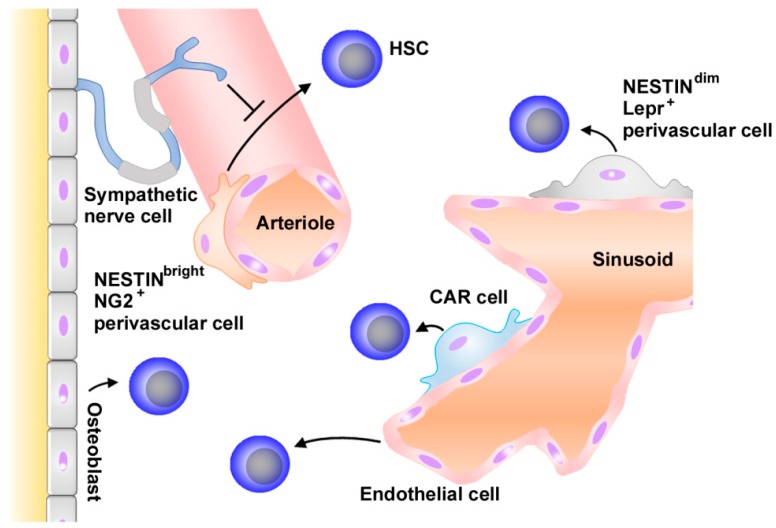
Schematic structure of hematopoietic niche. Hematopoietic stem cells (HSCs) are found adjacent to sinusoids and arterioles throughout the bone marrow. In sinusoids, endothelial cells, CXCL12-abundant reticular (CAR) cells, and Nestin^dim^Lepr^+^ perivascular cells promote HSC maintenance. Nestin^bright^NG^+^ perivascular cells adjacent to arterioles support HSCs. Sympathetic cells contribute to HSC maintenance by directly regulating CXCL12 expression by MSCs. Hematopoiesis can also be regulated by other types of cells such as osteoblasts. Arrows and “T” indicate stimulating and suppressive activities, respesctively.

**Figure 2:**
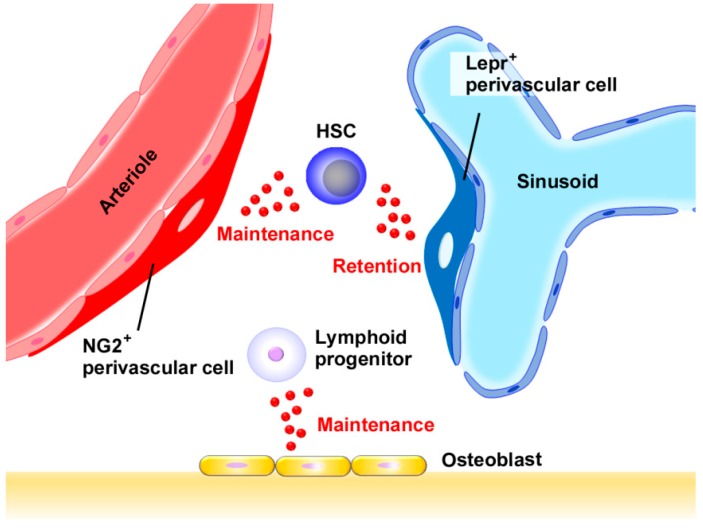
Presumed roles of CXCL12 in normal hematopoiesis. CXCL12 is produced by various types of cells in bone marrow, such as endothelial cells, mesenchymal stroma cells (MSCs), and CAR cells. stromal-derived factor (SDF), HSCs are maintained and retained by CXCL12 produced by Lepr^+^ perivascular cells in sinusoids or NG2^+^ perivascular cells in arterioles, while lymphoid progenitor cells are maintained by osteoblast-derived CXCL12. In addition, CXCL12 regulates the development of vasculature, which is crucial for hematopoietic functions.

HSCs are presumed to be localized in restricted areas in bone marrow, similarly observed on stem cells in other organs such as skin and intestine [13]. These restricted areas are called as niches, which are a functional unit to control stem cell number, fate, and behavior. Histological analysis on bone marrow tissue revealed that many HSCs were associated with sinusoidal endothelium in bone marrow, but some were associated with arterioles and endosteum (Figure 1) [1,14,15]. HSCs are mobile, regularly entering and existing in circulation [16]; therefore, it cannot be ruled out that these features can just mirror the transient presence of migrating HSCs in these areas. However, the sequential high-resolution imaging of mice indicated that HSCs trafficked to selected regions of bone marrow blood vessels abundantly expressing CXCL12 and E-selectin, and remained in these areas for weeks, eventually generating new cells [17]. These observations would indicate that perivascular and endosteal regions are hematopoietic niche and that CXCL12 has a crucial role by retaining HSCs in these niches (Figure 2).

WHIM (wart, hypogammaglobulinemia, recurrent infections, and myelokthexis) syndrome is an autosomal dominant combined primary immunodeficiency disease caused by loss of function mutations in CXCR4 [18]. WHIM patient-derived HSCs exhibited a remarkable competitive repopulating advantage over normal HSCs, without apparent deficits in engraftment [19]. These observations would indicate that CXCL12 could inhibit HSC proliferation. This notion can be corroborated by the observation that CXCL12 could in vitro inhibit entry of primitive hematopoietic cells into the cell cycle, and inactivation of its receptor CXCR4 in HSCs caused excessive HSC proliferation [20]. Moreover, the CXCR4^−/−^ HSCs were able to in vivo maintain a stable stem cell compartment and sustain hematopoiesis. Thus, the CXCR4–CXCL12 axis may be essential to keep HSC quiescence, thereby maintaining a lifelong steady pool of HSCs to sustain the highly regenerative hematopoietic system.

The localization of HSCs near blood vessels focused attention on endothelial cells as well as mesenchymal stroma cells (MSCs) that surround blood vessel throughout the bone marrow in terms of normal HSC physiology [1]. These MSCs are likely to be heterogeneous but can be defined as the cells expressing CD146 in humans [21], and SCF and CXCL12 were produced by MSCs as well as endothelial cells [3]. MSCs include Nestin^dim^Leptin Receptor-positive (Lepr^+^) perivascular cells adjacent to sinusoids and Nestin^bright^ nerve/glial antigen-positive 2 (NG2^+^) perivascular cells adjacent to arteiroles (Figure 1) [1,15]. Moreover, adipogenic progenitor cells with a reticular shape, abundantly express CXCL12 and consequently are named as CXCL12-abundant reticular (CAR) cells (Figure 1) [22]. Furthermore, many HSCs are in close contact with these CAR cells, suggesting that these CAR cells can also contribute to hematopoiesis.

Ding et al. reported that HSCs were depleted from bone marrow when the *SCF* gene was deleted from endothelial cells or Lepr^+^ perivascular cells but not hematopoietic cells, osteoblasts, or nestin-expressing cells [23]. They further demonstrated that most HSCs were lost when the *SCF* gene was deleted from both endothelial and Lepr^+^ perivascular cells. Thus, endothelial cells and perivascular cells can support HSC proliferation by producing a potent growth factor, SCF (Figure 1). Moreover, Asada et al. demonstrated that selective *CXCL12* gene deletion from arteriolar NG2^+^, but not from sinusoidal Lepr^+^ perivascular cells, reduced HSC and changed HSC localization in bone marrow [24]. They further observed that *SCF* gene deletion in Lepr^+^, but not that in NG2^+^ cells, reduced the HSC number in bone marrow. Thus, it is presumed that perivascular cells can differentially and cooperatively contribute to HSC maintenance in bone marrow.

The crucial roles of CXCL12 in normal hematopoiesis is evidenced by remarkable phenotypic changes in hematopoietic systems in the mice deficient in *CXCL12* or *CXCR4* genes [25,26,27,28], but these studies did not give a clear outline of which cell type has an indispensable role in hematopoiesis by producing CXCL12, because CXCL12 is primarily expressed by a wide variety of cells including perivascular MSCs, endothelial cells, CAR cells, osteoblasts, and some hematopoietic cells [29,30]. Several groups independently reported the effects of cell-type-specific *CXCL12* gene deletion on the HSC number and functions, but the results are not consistent, probably due to the incomplete gene deletion [24,29,30,31].

Omatsu et al. reported that short-term ablation of CAR cells in vivo impaired only the adipogenic and osteogenic differentiation potential of marrow cells [30]. However, HSCs from CAR cell-depleted mice were reduced in number and cell size, were more quiescent, and had increased expression of early myeloid selector genes, similar to the phenotype of wild-type HSCs cultured without a niche. Greenbaum et al. reported that deletion of the *CXCL12* gene from CAR cells and osteoblasts resulted in constitutive HPC mobilization and a loss of B-lymphoid progenitors, with normal HSC function [31]. Moreover, *CXCL12* gene deletion from endothelial cells resulted in a modest loss of long-term repopulating activity, and deletion of *CXCL12* from Prx1^+^ cells was associated with a marked loss of HSCs, long-term repopulating activity, HSC quiescence, and common lymphoid progenitors. Thus, CXCL12 expression in stromal cells in the perivascular region can support HSCs. Ding et al. reported that deletion of *CXCL12* from perivascular stromal cells depleted HSCs and certain restricted progenitors and mobilized these cells into circulation and that deletion of *CXCL12* from endothelial cells depleted HSCs but not myeloerythroid or lymphoid progenitors [29]. In contrast, deletion of *CXCL12* from osteoblasts depleted certain early lymphoid progenitors but not HSCs or myeloerythroid progenitors and did not mobilize these cells into circulation. However, deletion of *CXCL12* gene from hematopoietic cells or nestin-cre-expressing cells had little or no effect on HSCs or restricted progenitors. Asada et al. demonstrated that *CXCL12* or *SCF* gene deletion in all perivascular cells dramatically reduced HSCs and that the reduced NG2^+^ cell- but not Lepr^+^ cell-derived CXCL12 decreased HSC numbers and altered HSC localization in bone marrow [24]. Collectively, Lepr^+^ and NG2^+^ perivascular cells can maintain and retain HSCs cooperatively by producing CXCL12, while osteoblast-derived CXCL12 can mainly contribute to the maintenance of lymphoid progenitors [1,15] (Figure 2).

CXCL12 levels in bone marrow fluctuated in a circadian rhythm, through circadian adrenergic signals [32]. These adrenergic signals were locally delivered by nerves in the bone marrow and were transmitted to stromal cells by the β_3_-adrenergic receptor, leading to a decreased nuclear Sp1 transcription factor and the rapid downregulation of CXCL12. Depressed CXCL12 attenuated the retention of HSCs and their progenitors in bone marrow, culminating in the migration of these cells into circulation in a circadian rhythm. Thus, the nervous system may be able to regulate hematopoiesis through its effects on CXCL12, a master molecule for bone marrow niche functions (Figure 1).

### 2.2. Chemokines in Normal Hematopoiesis

Chemokines are a superfamily of chemotactic cytokines consisting of more than 40 structurally related molecules (Table 1) [33]. Chemokines exert their biological functions through binding their cognate 7-transmembrane spanning receptors coupled with heterotrimeric GTP-binding proteins [33]. Thus, the target cell specificity of each chemokine is determined mainly by the expression pattern of its corresponding receptor. Chemokines exhibit a similar three-dimensional structure [34]. Triple-stranded β sheets are shaped by two intramolecular disulfide bonds, which are formed between the first and third cysteines and between the second and fourth cysteines. The carboxy-terminal region forms an α-helix with the capacity to bind heparin, which accounts for the ability of chemokines to bind to proteoglycans and glycosaminoglycans with a high avidity. Consequently, most chemokines are produced as secretory proteins, but once being secreted, they can be immobilized on endothelium and extracellular matrix by interacting with proteoglycans and glycosaminoglycans [34]. The immobilization facilitates the generation of a concentration gradient, which is crucial for inducing the target cells to migrate in a directed way.

Chemokines are divided into four subclasses; CXC, CC, CX_3_C, and C (Table 1) [33]. The first cysteines are separated by one and three amino acids in CXC and CX_3_C chemokines, respectively, while the first two cysteines are adjacent in CC chemokines. The single C chemokine does not possess the second and fourth cysteines. Systemic nomenclature is based on their cysteine subclass roots, followed by “L” for ligand and the numbers correspond to the number used in the corresponding gene nomenclature. As most chemokine receptors can bind to a single chemokine subclass, the nomenclature system of chemokine receptors is rooted by the chemokine subclass specificity, followed by “R” for receptor and the corresponding number. The CXC chemokines can be further grouped on the presence or the absence of a 3-amino acid sequence, glutamic acid-leucine-arginine (the “ELR” motif), immediately preceding the CXC sequence [35]. In general, CXC chemokines with the ELR motif exhibit a neutrophil chemotactic and an angiogenic activity by binding with their specific receptors, CXCR1 and/or CXCR2.

Based on their expression pattern, chemokines can be arbitrarily classified as inflammatory or homeostatic [36]. Inflammatory chemokines are abundantly expressed under various inflammatory conditions and can regulate the infiltration of inflammatory cells including granulocytes and monocyte/macrophages. Representative inflammatory chemokines include CXC chemokines with the ELR motif with a potent neutrophil chemotactic activity and CCL2 with a potent monocyte/macrophage chemotactic activity. In contrast, homeostatic chemokines are expressed constitutively in specific tissues or cells, and can regulate organogenesis of various organs.

CXCL12 is a representative homeostatic chemokine and is expressed constitutively in normal bone marrow [25]. As we discussed in the previous section, CXCL12 is expressed in diverse types of cells in adult bone marrow, including osteoblasts, endothelial cells, nestin-expressing cells, and adipogenic progenitor cells, CAR cells. Moreover, CXCL12 has overlapping but distinct impacts on hematopoiesis, by retaining HSCs in the bone marrow niche [17] and inhibiting cell cycle entry of HSCs to keep the pool of quiescent HSCs [20] (Figure 2). In addition to its direct effects on the bone marrow niche and HSCs, CXCL12 can indirectly have impacts on hematopoiesis. The defect of vessel development in mice lacking CXCR4 [26] indicates that the CXCL12–CXCR4 axis may be able to indirectly affect hematopoiesis, by regulating the development of vasculature, which can have a crucial effect on the hematopoietic niche residing near vasculature (Figure 1).

The HSC pool in bone marrow is stably maintained by an intricate balance between differentiation, self-renewal, and reversible quiescent cell cycle arrest of HSCs. During the 1970s and 1980s, a molecule present in bone marrow microenvironment has been presumed to regulate steady-state quiescent arrest of HSCs and has been named a stem cell inhibitor (SCI) (Table 1) [37]. One candidate for this stem cell inhibitor can be CXCL12, since CXCL12 can in vitro inhibit entry of primitive hematopoietic cells into the cell cycle, and inactivation of its receptor CXCR4 in HSCs causes excessive HSC proliferation [20].

**Figure 3:**
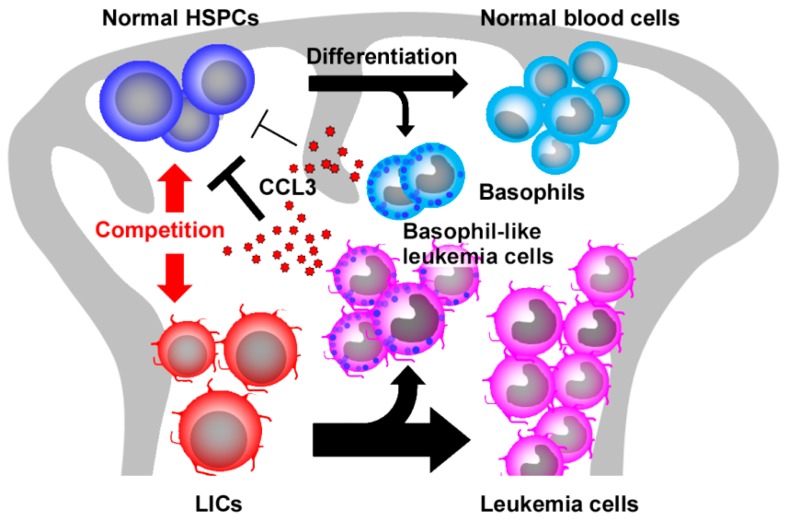
Presumed roles of CCL3 in chronic myeloid leukemia (CML). A small amount of CCL3 with a potent inhibitory activity for normal HSPCs is produced constitutively by basophils under normal circumstances. In CML bone marrow, a large number of basophil-like leukemia cells are generated from LICs and produce abundantly CCL3, which inhibits normal HSPC proliferation to confer growth advantage to LICs over normal HSPCs. Black arrows indicate the differentiation pathways while the red arrow indicate the competition between normal HSCs and LICs. “T” indicates the suppressive activities.

Another candidate for SCI is CCL3 because it can reversibly inhibit colony formation and proliferation of hematopoietic stem/progenitor cells (HSPCs) both in vitro and in vivo [38,39,40]. Moreover, CCL3 can maintain a quiescent state in HSCs by blocking cell cycle entry [41]. In contrast, CCL3 can activate the proliferation of more mature progenitor cells [39,42]. Indeed, CCL3 was expressed constitutively by normal basophils present in normal bone marrow [43]. Moreover, the genetic deletion of *CCL3* gene in donor cells caused exaggerated reconstitution of donor-derived hematopoietic cells upon bone marrow transplantation. Furthermore, deletion of basophils recapitulated similar phenotypes upon bone marrow transplantation [43]. Thus, CCL3 produced by basophils may be able to act as SCI to dampen the proliferation of normal HSPCs (Figure 3).

There are more than 20 chemokines that have been found to suppress HSPC proliferation in a manner similar to that of CCL3 (Table 1) [44,45]. The list crosses the CC, CXC, and C subfamily and includes CXCL2, CXCL4, CXCL5, CXCL6, CXCL8, CXCL9, CXCL10, CCL1, CCL2, CCL6, CCL9/10, CCL11, CCL12, CCL13, CCL15, CCL16, CCL18, CCL19, CCL20, CCL21, CCL23, CCL24, CCL25, and XCL1. However, except CXCL12 and CCL3, the suppressive effects of these chemokines have not yet been examined extensively.

Granulocyte colony-stimulating factor (G-CSF) has been clinically used to mobilize HSPCs into peripheral blood and to use them for hematopoietic transplantation [46]. This success spurred the investigation on the ability of other cytokines and chemokines to mobilize HSPCs into peripheral blood. In fact, CCL3 preferentially mobilized the more primitive progenitors with marrow repopulating activity, releasing four times the number achieved with G-CSF alone [47], by interacting mainly with CCR1 but not CCR5, among its specific receptors [48]. Multiple ligands for CXCR2, CXCL1, CXCL2, and CXCL8 also rapidly mobilized HSPCs with long-term repopulating ability into peripheral blood in mice and monkeys [49,50,51,52]. However, CXCR2 was not expressed on HSC and HPC [53]. Detailed analysis indicated that neutrophils were a common target for HSPC mobilization induced by CXCR2 ligands and G-CSF and that this mobilization was mediated by neutrophil-derived plasma matrix metalloproteinase (MMP)-9 [52]. However, how MMP-9 can mobilize HSPCs still remains elusive. 

A CXCR4 agonist [54] and a CXCR4 antagonist [55] have similarly mobilized alone or synergistically with G-CSF. These apparently puzzling effects can be explained by depressed CXCR4 expression by HSPCs by the treatment with a CXCR4 agonist or antagonist. Considering the crucial roles of the CXC12–CXCR4 axis in HSPC maintenance in bone marrow [25,26,27,28], depressed CXCR4 expression on HSPCs may be able to reduce the interaction between HSPCs and CXCL12-expressing niche and subsequent HSPC migration from bone marrow.

The crucial involvement of the CXCL12–CXCR4 axis in normal hematopoiesis is substantiated by remarkable phenotypic changes in hematopoietic systems in the mice deficient in CXCL12 or CXCR4 [25,26,27,28]. Among mice deficient in other chemokine and chemokine receptor genes, mice deficient in either CCL5 or atypical chemokine receptor (ACKR)1, also known as Duffy antigen receptor for chemokines (DARC) [56], exhibited abnormalities in HSCs. Enhanced CCL5 expression in aging stem cell milieu was associated with myeloid skewing and reciprocally, CCL5-deficient mice exhibited a decrease in myeloid-biased HSCs and an increase in T cell and lymphoid-biased HSCs in a cell-autonomous manner [57]. These observations suggested that aging-related changes in hematopoietic functions can arise from aging-related augmented CCL5 expression in bone marrow.

ACKR1 is a sink receptor for various chemokines because it can bind various chemokines including both CXC and CC chemokines without transducing any intracellular signal [56]. Nucleated erythroid cells expressed abundantly ACKR1, which facilitated their direct contact with HSCs, and the absence of ACKR1 decreased HSPCs and alters their differentiation with aberrant neutrophil dysfunction [58]. Of interest is that neutrophil abnormalities were observed in Duffy antigen-negative persons. However, it is an open question which chemokine(s) is involved in phenotypic changes observed in ACKR1 deficiency.

### 2.3. CML

CML is a myeloproliferaive disorder arising from the malignant transformation of HSCs in chronic phases or of HPCs in the blast crisis phase [4]. It harbors a characteristic chromosomal abnormality, the Philadelphia chromosome, which results from a reciprocal translocation between the long arms of chromosome 9 (ch9) and 22 (ch22) [59]. t (9,22) causes the juxtaposition of the *ABL-1* gene, the human analogue of the *v-ABL* oncogene, from ch9 with the *BCR* housekeeping gene on ch22 to produce the fusion of the *BCR-ABL* gene, which can be translated to BCR-ABL protein. *ABL-1* encodes a non-receptor tyrosine kinase that phosphorylates substrate proteins through its SH1 domain, and eventually has profound impacts on crucial cellular activities, leading to increased cell proliferation and resistance to apoptosis [60]. When *BCR-ABL* fusion gene is generated, the upstream control element of the *ABL-1* gene is deleted and is replaced with the *BCR* gene. As a consequence, BCR-ABL protein is capable of phosphorylating constitutively itself, resulting in uncontrolled activation of a wide variety of downstream effector pathways and the transformation of HSCs into LSCs, which can behave in leukemogenic hematopoiesis, as do HSCs in normal hematopoiesis. Moreover, CML cells depend on their own BCR-ABL’s constitutive tyrosine kinase activity for their autonomous proliferation, and BCR-ABL protein is selectively expressed in CML cells but not normal cells including hematopoietic cells. Due to these characteristics, BCR-ABL protein has been a preferable druggable target [59,60].

The clinical application of imatinib, a relatively selective inhibitor for BCR-ABL’s tyrosine kinase, has transformed CML, a thitherto intractable disease, into a controllable one [59]. Subsequent development of additional tyrosine kinase inhibitors (TKIs) further improved the prognosis of CML patients. However, a long-term treatment with TKIs frequently fails to eradicate CML cells; as a consequence, the discontinuation of TKIs can cause CML recurrence even if *BCR-ABL* gene expression cannot be detected for a considerable period with the most sensitive molecular biological methods [61]. BCR-ABL can promote LSC and their progeny survival through its kinase-independent activities such as modulation of Hedgehog and BRCA1 pathways, the activities that cannot be inhibited by TKIs [62]. This may account partly for the failure of TKIs to eradicate LSCs and eventually cure CML. In addition to these leukemia cell-autonomous mechanisms, the resistance to TKIs can arise from the effects of various factors, which are produced by bone marrow microenvironments to help LSCs survive TKIs, and TGF-β can be one of the growth factors that can confer growth advantage on LSCs during TKI treatment [63].

At both the initiation phase and the TKI-induced remission stage of CML, a small number of BCR-ABL-expressing LSCs are present at one site in bone marrow, where normal hematopoietic cells including hematopoietic stem/progenitor cells (HSPCs) are abundant [62]. LSCs are presumed to compete for hematopoietic niche with normal hematopoietic cells in the primary bone marrow site. After LSCs predominate over normal hematopoietic cells to occupy one bone marrow site, they can infiltrate other bone marrow sites and the spleen, and they can finally appear in peripheral blood as they differentiate into the leukemic cells with more mature phenotypes. The analysis on mathematical models, which were constructed by using Bayesian inference techniques, supported the notion that clinical outcome in CML could be determined by the competition over niche between normal HSPCs and LSCs [64].

Simultaneously, evidence is accumulating to indicate that LSC maintenance and subsequent CML development require the cues provided by the bone marrow niche [62]. In fact, TGF-β and its related molecules, bone morphogenetic protein 2 (BMP2) and BMP4, were expressed by the bone marrow niche, and can promote CML cell expansion [65,66]. Moreover, homing and engraftment of BCR-ABL-expressing LSCs required E-selectin expression by the bone marrow endothelial niche [67].

Collectively, although BCR-ABL can confer the autonomous growth capacity on LSCs, subsequent CML development and the responsiveness to drugs can be markedly modulated by signals provided by bone marrow microenvironments, particularly the hematopoietic niche.

### 2.4. CXCL12 in CML

In transgenic mice with inducible *BCR-ABL* gene, its induction can cause chronic phase CML [68]. In this CML model, LSCs were restricted to cells with long-term hematopoietic stem cell (LTHSC) phenotype. CML LTHSC demonstrated reduced homing and retention in the bone marrow and increased egress to spleen. These changes were associated with decreased CXCL12 expression in bone marrow and reciprocally increased CXCL12 expression in the spleen [68]. In line with these observations, CML cell-derived exosome delivered miR-126 to reduce CXCL12 expression in endothelial cells [69]. Of interest is that CML LSCs were characterized by a specific expression of a surface expression of dipeptidylpeptidase-IV, which can cleave CXCL12 and reduce its biological activities [70]. Thus, CML cells may be able to reduce functional CXCL12 expression in the cells surrounding them, to facilitate their egress from bone marrow to other sites including spleen and peripheral blood.

Depressed CXCL12 expression in CML bone marrow can arise from an additional mechanism. CML bone marrow exhibited increased levels of several cytokines and chemokines including IL-1α, IL-1β, IL-6, G-CSF, TNF-α, CCL3, and CCL4 [68]. Among these cytokines and chemokines, only G-CSF in vitro decreased CXCL12 expression in bone marrow stromal cells, while anti-G-CSF antibody treatment increased CXCL12 expression and CML LSC numbers in bone marrow, and reciprocally decreased CML LSC numbers in the spleen [68]. Similar changes were observed when CML mice are treated with a TKI, imatinib. Moreover, human CML bone marrow exhibited decreased CXCL12 and increased G-CSF expression [68]. These observations would indicate that CML cells, particularly LSCs, can be retained in bone marrow by the action of CXCL12, which may be under the control of the cytokine network consisting of G-CSF and other pro-inflammatory cytokines. Moreover, reduced CXCL12-mediated signals may be able to induce the egress of CML cells, particularly LSCs, to extra-bone marrow sites, thereby promoting CML progression.

CXCL12 may be able to elicit similar concentration-dependent growth suppressive effects on normal and CML CD34^+^ cells in colony-forming cell assays, but no significant differences were observed on CXCR4 expression and responsiveness to CXCL12-induced increases in intracellular calcium levels between normal and CML CD34^+^ cells [71]. However, human CD34^+^ CML cells in peripheral blood showed a reduced migratory response to CXCL12, compared with human normal CD34^+^ cells [71]. Moreover, human BCR-ABL^+^CD34^+^CXCR4^+^ cells displayed reductions in CXCL12-induced integrin-dependent polarization and migration, and binding to vascular cell adhesion molecule-1 or fibronectin, compared with normal CD34^+^CXCR4^+^ cells [72]. Comprehensive gene expression analysis revealed depressed CXCR4 mRNA expression in CML CD34^+^ cells, compared with normal CD34^+^ cells, but the study did not examine the cell surface expression levels [73]. Chang and colleagues, however, proposed that the impaired chemotactic response of CML CD34^+^ cells to CXCL12 might be due to an intracellular signaling defect downstream of the receptor. This notion may be supported by the observations that BCR-ABL could persistently activate Cdc42 GTPase, which is crucially involved in cytoskeletal remodeling and directional sensing, and that its sustained activation reduced CXCL12-mediated chemotactic response by desensitizing the actin polarization required for directional migration [74].

Evidence is accumulating to indicate that LSCs are rather resistant to various chemotherapeutic drugs and TKIs through the interaction with hematopoietic niche [75]. The CXCL12–CXCR4 axis may have profound effects on the interaction of CML cells with hematopoietic niche consisting of extracellular matrix and stromal cells in bone marrow. A TKI, imatinib, enhanced CXCR4 expression in CML cells and caused the migration of CML cells to the CXCL12-expressing bone marrow microenvironment niche, which provided CML cells with anti-apoptotic signals and subsequently resistance to imatinib [76]. It also promoted CXCR4 redistribution in CML cells into the lipid raft fraction, where it co-localized with active phosphorylated form of a src-related kinase, Lyn [77]. Through these compartmental changes of multivalent CXCR4 and activated Lyn complexes, CML cells migrated to the bone marrow niche, where they survived despite the presence of imatinib. Likewise, the resistance to another TKI, nilotinib, was associated with CXCR4-mediated adhesion of CML cells to extracellular matrix components and bone marrow stromal cells [78]. These observations would indicate that the CXCL12–CXCR4 axis could prevent CML cells from apoptosis by enhancing the interaction of these cells with hematopoietic niche, which could confer growth-promoting and/or anti-apoptotic signals.

CXCR4 directly delivered growth-promoting and/or anti-apoptotic signals to CML cells. CXCR4 activated its downstream PI3K/Akt signal pathway and promoted the translocation of NF-κB complexes into nucleus in CML cells, thereby decreasing the expression of apoptosis-related molecules [79]. CXCL12 conferred the resistance to adriamycin on CML cells by augmenting their CXCR4 expression and CXCR4 siRNA treatment can partially reduce adriamycin resistance [79]. Moreover, CXCL12 activated pro-survival signal pathways including extracellular signal-regulated kinase (Erk)-1/2, Akt, S-6-kinase, STAT3, and STAT5, and in vitro treatment with a CXCR4 antagonist directly inhibited cell growth and induced eventually cell death [80]. Furthermore, CXCL12 increased the expression of ABC transporters including multidrug resistance 1, ATP-binding cassette subfamily C member 1 (ABCC1), and ATP-binding cassette subfamily G member 2 (ABCG2) [81], and their enhanced expression might substantially contribute to resistance to chemotherapeutic agents by exporting chemotherapeutic drugs outside cells.

The roles of the CXCL12–CXCR4 axis in CML cell survival prompted several groups to examine the therapeutic efficacy of CXCR4 antagonists for CML in preclinical models. A CXCR4 antagonist, plerixafor, had no effects on CML progression in mice intravenously injected with BCR-ABL-transduced mouse 32D cells, but its combination with nilotinib significantly delayed time to relapse, and significantly prolonged survival when compared with nilotinib monotherapy, without any significant effects on the body weights of the mice [78]. Another CXCR4 antagonist, BKT140, effectively reduced the growth of the tumors arising from subcutaneous injection of a human CML cell line, K562, and its combination with imatinib essentially abrogated tumor growth, resulting in 95% reduction in tumor size and weight, together with extensive necrotic tissue damage [80]. However, these models used a cloned cell line as a source of CML cells, so the observations need to be extrapolated with caution. When CML was induced by bone marrow transplantation with BCR-ABL-expressing hematopoeitic cells, the combined treatment with plerixafor and TKIs failed to reduce leukemia burden over TKIs alone [82]. Moreover, mice receiving plerixafor had an increased incidence of neurologic symptoms in association with BCR-ABL-expressing cell infiltration into the central nervous system, probably arising from a potent capacity of a CXCR4 antagonist to induce the egress of CML cells from bone marrow [82]. Thus, clinical application of a CXCR4 antagonist warrants more detailed and meticulous investigation.

### 2.5. CCL3 and Its Related Chemokines in CML

The identification of CCL3 as SCI incited several groups to examine its effects on CML cell proliferation. Both TGF-β_1_ and CCL3 inhibited colony forming unit (CFU)-A colony formation from normal hematopoietic cells, whereas TGF-β_1_ but not CCL3 inhibited CFU-A colony formation from CML progenitor cells [83]. Likewise, the CML cell population exhibited an increased rate of turnover at primitive clonogenic cell levels, due to an inability of CML cells to respond to the cytostatic effects of CCL3 [84]. Moreover, the addition of CCL3 to normal long-term cultures of hematopoietic cells reversibly and specifically blocked the activation of primitive progenitor with high proliferative potential but not mature progenitor with lower proliferative potential [85]. On the contrary, CCL3 failed to block the cell cycling of primitive CML progenitors, when it was added to similar long-term cultures containing normal bone marrow adherent cell layers but supporting CML hematopoiesis.

Nicholls et al. reported that CCL3 binding sites were reduced on CML CD34^+^ cells, compared with normal CD34^+^ population and proposed that reduced CCL3 binding sites were responsible for reduced responsiveness of CML progenitor cells to CCL3 [86]. Chasty et al., however, indicated the presence of a single population of cells that expressed cell surface receptors for CCL3 independently of cell cycle status [87]. They further proposed that CML progenitor cells might be refractory to CCL3 as a result of events downstream from receptor expression. Consistently, Dürig et al. reported that normal and CML CD34^+^ cells bound to biotinylated CCL3 to similar extents [88]. They further demonstrated that a specific receptor for CCL3, CCR5, was expressed at comparable levels in normal and CML CD34^+^ cells. Our analysis on the expression of CCL3 receptors, CCR1 and CCR5, revealed that CCR1 and to a markedly lesser degree, CCR5, was expressed on normal lineage marker (lin)^−^c-kit^+^ progenitor cells and that CCR1 but not CCR5 expression was maintained by CML lin^−^c-kit^+^ progenitor cells [89]. Thus, decreased CCL3 receptor expression on CML progenitor cells cannot explain the inability of these cells to respond to CCL3.

This assumption is supported by the observations by Wark et al. [90]. They established a cell line which can express temperature-sensitive *ABL* gene, from a growth factor-dependent multipotent stem cell line, in which growth was normally suppressed by CCL3. When ABL expression was induced, the cells lost the capacity to respond to CCL3 [90]. They further demonstrated that ABL expression did not change the number and affinity of CCL3 binding sites on the cells but did abrogate CCL3-mediated increases in intracellular Ca^2+^ concentrations. Thus, it is likely that ABL’s tyrosine kinase activity can directly reduce the responsiveness to CCL3.

Murine CML-like disease can be induced by transferring human-derived *BCR-ABL* gene-transduced primitive bone marrow cells intravenously to a lethally irradiated host [91]. This model has been widely used, but in this model, lethal irradiation completely destructs the normal hematopoietic microenvironment to enable BCR-BAL^+^ leukemic cells to home to the bone marrow and grow therein. Thus, it is difficult to elucidate the role of bone marrow microenvironment in CML development, particularly at its early phase. The other model utilizes an inducible *BCR-ABL* transgenic mouse, which can express *BCR-ABL* gene in HSPCs in an inducible manner [92]. However, in this model, it is not easy to selectively tag leukemia cells for the examination of leukemia cell trafficking. In order to circumvent these problems, we established a novel CML model, by transferring human-derived *BCR-ABL* gene-transduced primitive bone marrow cells to the bone marrow of non-irradiated nude mice [89].

In this model, the expression of CCL3 but not those of other chemokines were increased [89], in contrast to the previous report that in addition to CCL3, CCL4, and CXCL12 expression was enhanced in bone marrow of inducible *BCR-ABL* transgenic mice, which developed CML [68]. CCL3-deficient BCR-ABL-transduced HSPCs failed to induce CML-like phenotypes when injected into the bone marrow of non-irradiated host, but induced CML-like phenotypes when transferred intravenously into irradiated hosts [89]. Thus, CCL3 might have a role in CML development, only when bone marrow microenvironment can be preserved. Consistently, normal HSPCs directly impeded the maintenance of LSCs when these cells did not express either CCR1 and/or CCR5, the specific receptors for CCL3 [89]. The potential roles of the interplay between CCL3 and its receptors may be further supported by the observation that a CCR5 antagonist prevented CML development in nude mice receiving intra-bone marrow injection of BCR-ABL^+^ leukemia cells, when it was initiated immediately after the injection [43].

Detailed analysis on this model identified basophil-like leukemia cells and to a lesser degree, normal basophils as a source of CCL3 in CML bone marrow [43]. Similar observations were obtained on the bone marrow of patients with CML. Moreover, deletion of basophil-like leukemia cells recapitulated the phenotypes observed when CCL3-deficient BCR-ABL-transduced HSPCs were injected into the bone marrow of non-irradiated host [43]. Collectively, basophilia, a frequently observed hematological abnormality in CML patients, may not simply be an accompanying phenomenon but may have a pathogenic role in CML development, by producing abundantly CCL3, which can dampen normal HSPC but not LSC proliferation, and eventually provide LSCs with a growth advantage over normal HSPCs, particularly at the early phase of CML development (Figure 3). Thus, basophils and/or the CCL3 axis can be a novel target for CML treatment.

CCL3 may cause myeloproliferative neoplasm (MPN) in a different manner. About half of patients with Noonan syndrome harbor a germline activating mutations of the protein tyrosine phosphatase src homology-2 domain containing protein tyrosine phosphatase (SHP2) (encoded by *PTPN11*), a positive regulator of the RAS signaling pathway [93]. These patients have an increased risk of developing leukemia, especially juvenile myelomonocytic leukemia (JMML), a childhood MPN. *Ptpn11* mutations in mesenchymal stem/progenitor cells and osteoprogenitors, but not in differentiated osteoblasts or endothelial cells, caused excessive production of CCL3, which recruited monocytes to the area where HSCs also reside [94]. Consequently, HSCs were hyperactivated by monocyte-derived interleukin-1β and other proinflammatory cytokines, leading to the development of MPN, particularly donor-cell-derived MPN following stem cell transplantation [94]. Thus, bone marrow stroma-derived CCL3 may be able to cause leukemogenesis by inducing infiltration of monocytes with a capacity to produce abundantly various pro-inflammatory cytokines and to eventually enhance the proliferation of pre-leukemic cells.

### 2.6. Other Chemokines in CML

Evidence is accumulating to indicate the involvement of other chemokines in CML pathogenesis. CXCL8 protein expression was enhanced by BCR-ABL expression and was inhibited by TKIs such as dasatinib and nilotinib, implicating CXCL8 as a useful marker for the monitoring of CML inhibitor efficacy [95]. The analysis on a human CML cell line, LAMA84, revealed that CML cell-derived exosomes stimulated bone marrow stromal cells to produce CXCL8 that, in turn, was able to enhance the capacity of CML cells to migrate and to adhere to stromal cells, and to promote CML cell proliferation both in vitro and in vivo [96]. However, the effects of CXCL8 on other CML cell lines in general remain elusive.

CCL2 blocked the cell cycle entry of normal HSPCs but not CML progenitor cells, whereas TGF-β inhibited cell cycle entry of both normal HSPCs and CML LSCs [97]. Of interest is that comprehensive analysis revealed enhanced CCL2 but reduced TGF-β expression in CML LSCs, compared with normal HSPCs [98]. Thus, CCL2 and CCL3 can contribute to CML development in similar manners.

*BCR-ABL* gene transduction into umbilical cord blood-derived CD34^+^ cells suppressed the expression of a chemokine receptor, CCR7, as well as adhesion molecules such as L-selectin and intercellular adhesion molecule (ICAM)-1 [99]. Moreover, depressed CCR7 expression was associated with reduced migratory ability to its ligands, CCL19 and CCL21. On the contrary, Kubo et al. observed that BCR-ABL cooperated with signal transducing adaptor protein (STAP)-2 to induce the expression of CCR7 and CXCR4 and the production of the ligands for CCR7, CCL19, and CCL21, in a murine hematopoietic BaF/3 cell [100], raising the possibility of the contribution of the CCR7 axis to CML cell proliferation. Hromas et al., however, claimed that CCR7 ligands, CCL19 and CCL21, and a CCR6 ligand, CCL20, inhibited proliferation of CML progenitor cells as well as normal progenitor cells [101]. Collectively, the roles of the CCR7 axis in CML pathogenesis remain an open question.

## 3. Future Perspective

The identification of *BCR-ABL* gene as a driver mutation for CML has spurred the development of TKIs targeting the tyrosine kinase activity of BCR-ABL fusion protein and the clinical application of TKIs has remarkably improved the prognosis of CML patients [59,60]. TKIs mainly target leukemia cells in cell cycle but LSCs usually stay dormant through the interaction with the bone marrow microenvironment, particularly the niche [75]. As a consequence, even long-term treatment with TKIs frequently fail to eradicate LSCs and to eventually cure CML [62]. Thus, in order to develop a novel therapeutic strategy for CML, it is necessary to elucidate the cellular and molecular mechanisms underlying the interaction between LSCs and bone marrow microenvironments.

As we discussed here, evidence is accumulating that indicates the crucial roles of chemokines, particularly CXCL12 and CCL3, in CML progression [77,89]. More detailed analysis on the roles of these chemokines can enable the development of a novel therapeutic strategy to supplement the treatment with TKIs.

## Figures and Tables

**Table 1:** Summary of chemokines and their receptors.

Standard Name	Common Alias	Receptor	SCI Activity
CXCL1	groα, melanoma growth stimulating activity (MGSA), KC	CXCR2	
CXCL2	groβ, macrophage inflammatory protein (MIP)-2α	CXCR2	+
CXCL3	groγ, MIP-2β	CXCR2	
CXCL4	platelet factor-4 (PF-4)	CXCR3	+
CXCL5	epithelial neutrophil activating peptide (ENA)-78	CXCR2 > CXCR2	+
CXCL6	granulocyte chemotactic protein (GCP)-2	CXCR1, CXCR2	+
CXCL7	neutrophil activating protein (NAP)-2	CXCR2	
CXCL8	interleukin-8 (IL-8)	CXCR1, CXCR2	+
CXCL9	monokine induced by interferon γ (Mig)	CXCR3	+
CXCL10	interferon inducible protein (IP)-10	CXCR3	+
CXCL11	interferon inducible T-cell α chemoattractant (I-TAC)	CXCR3, CXCR7	
CXCL12	stromal-derived factor (SDF)-1	CXCR4, CXCR7	+
CXCL13	B lymphocyte chemoattractant (BLC)	CXCR5	
CXCL14	breast and kidney expressed chemokine (BRAK)	?	
CXCL15	lungkine	?	
CXCL16	scavenger receptor for phosphatidylserine and oxidized lipoprotein (SR-PSOX)	CXCR6	
CXCL17		?	
CCL1	I-309	CCR8	+
CCL2	monocyte chemoattractant (MCP)-1	CCR2	+
CCL3	MIP-1α	CCR1, CCR5	+
CCL4	MIP-1β	CCR5 > CCR1	
CCL5	regulated upon activation normal T cell expressed and secreted (RANTES)	CCR1, CCR5, CCR3	
CCL6	C10, macrophage inflammatory protein-related protein (MRP)-1	?	+
CCL7	MCP-3	CCR1,CCR2, CCR3 > CCR5	
CCL8	MCP-2	CCR2, CCR1, CCR3, CCR5	
CCL9	MRP-2, MIP-1γ	?	+
CCL10		?	+
CCL11	eotaxin	CCR3 > CCR5	+
CCL12	MCP-5	?	+
CCL13	MCP-4	CCR1,CCR2, CCR3 > CCR5	+
CCL14	hemofiltrate CC chemokine (HCC)-1	CCR1	
CCL15	HCC-2	CCR1, CCR3	+
CCL16	HCC-4	CCR1, CCR2, CCR5	+
CCL17	thymus and activation-regulated chemokine (TARC)	CCR4 > CCR8	
CCL18	pulmonary and activation-regulated chemokine (PARC)	?	+
CCL19	EBI1-ligand chemokine (ELC)	CCR7	+
CCL20	MIP-3α, liver and activation-related chemokine (LARC)	CCR6	+
CCL21	secondary lymphoid chemokine (SLC)	CCR7	+
CCL22	macrophage-derived chemokine (MDC)	CCR4	
CCL23	myeloid progenitor inhibitory factor-1 (MPIF-1)	CCR1	+
CCL24	eotaxin-2	CCR3	+
CCL25	thymus-expressed chemokine (TECK)	CCR9	+
CCL26	eotaxin-3	CCR3	
CCL27	cutaneous T-cell attracting chemokine (CTACK)	CCR10	
CCL28	mucosae-associated epithelial chemokine (MEC)	CCR3, CCR10	
XCL1	lymphotaxin-α	XCR1	+
XCL2	lymphotaxin-β	XCR1	+
CX3L1	fractalkine	CX3CR1	

“?” in receptor column indicates that the receptor is unidentified; “+” in SCI activity column indicate that the chemokine can exhibit SCI activity.
